# The influence of initial developmental status on the life-history of sea trout (*Salmo trutta*)

**DOI:** 10.1038/s41598-019-49175-0

**Published:** 2019-09-17

**Authors:** Diego del Villar-Guerra, Martin H. Larsen, Henrik Baktoft, Anders Koed, Kim Aarestrup

**Affiliations:** 1 Loughs Agency, 22 Victoria Road, Londonderry, BT472AB, Northern Ireland UK; 2 Danish Centre for Wild Salmon, Brusgårdsvej 15, 8960 Randers, SØ Denmark; 3 Technical University of Denmark, National Institute of Aquatic Sciences, Denmark, Vejlsøvej 39, 8600 Silkeborg, Denmark

**Keywords:** Animal migration, Behavioural ecology

## Abstract

Spring migrating sea trout juveniles can be classified as parr, pre-smolt or smolt based on body morphology and osmoregulatory capacity. In this respect, parr are assumed to be less prepared for a marine life and to have lower survival at sea than pre-smolts and smolts. However, the behaviour and survival of these trout phenotypes upon entering the sea is not well known. Using passive integrated transponder telemetry, this study found that the return rate from the sea to the natal river was higher for parr compared to pre-smolts and smolts. Additionally, trout classified as parr generally migrated earlier to the sea and a larger proportion returned to the river after less than one year at sea. The daily mortality rate at sea was comparable among the different phenotypes of trout, suggesting that the higher proportion of returning parr to the river was linked to their shorter duration at sea. These results provide evidence of different life-history strategies for seaward-migrating juvenile sea trout, ultimately affecting their return rate to the natal river. Investigations failing to consider downstream migrating parr and pre-smolts risks neglecting a large part of the anadromous population and may result in inaccurate assessments of sea trout stocks in rivers.

## Introduction

The life-cycle of anadromous trout (sea trout; *Salmo trutta*) involves a series of seasonal movements between freshwater and marine habitats^1^. The shift from freshwater to saltwater is seen as an adaptive strategy allowing migratory individuals to attain larger sizes and greater reproductive potential^2,3^. However, migration is energetically demanding and usually associated with a high loss of individuals in the first period at sea^4–8^. Little is known about factors influencing sea survival in wild sea trout, apart from predation^9,10^. As many wild sea trout populations are declining all over Europe^11^, there is currently growing interest in understanding factors affecting marine survival to improve the management of wild sea trout populations^12^.

Juvenile trout typically migrate to the sea in the spring and early summer (e.g.^13–15^). In Denmark, this migration to sea usually takes place from early March to the beginning of June with a peak in number of migrants in the last half of April and early May^16–18^. The transition from freshwater to saltwater involves a series of complex, interrelated processes including physiological, morphological and behavioural changes that optimize performance and survival in the marine environment, known as smoltification^19,20^. By completion of smoltification, trout can regulate the ion concentration of their body fluid to cope with saltwater^21,22^. The age of seaward-migrating trout in Denmark range from one to four years^16,17^, and the average length of trout smolts varies between 120 and 200 mm, with a range from 70 to 300 mm^16,23^.

**Figure 1:**
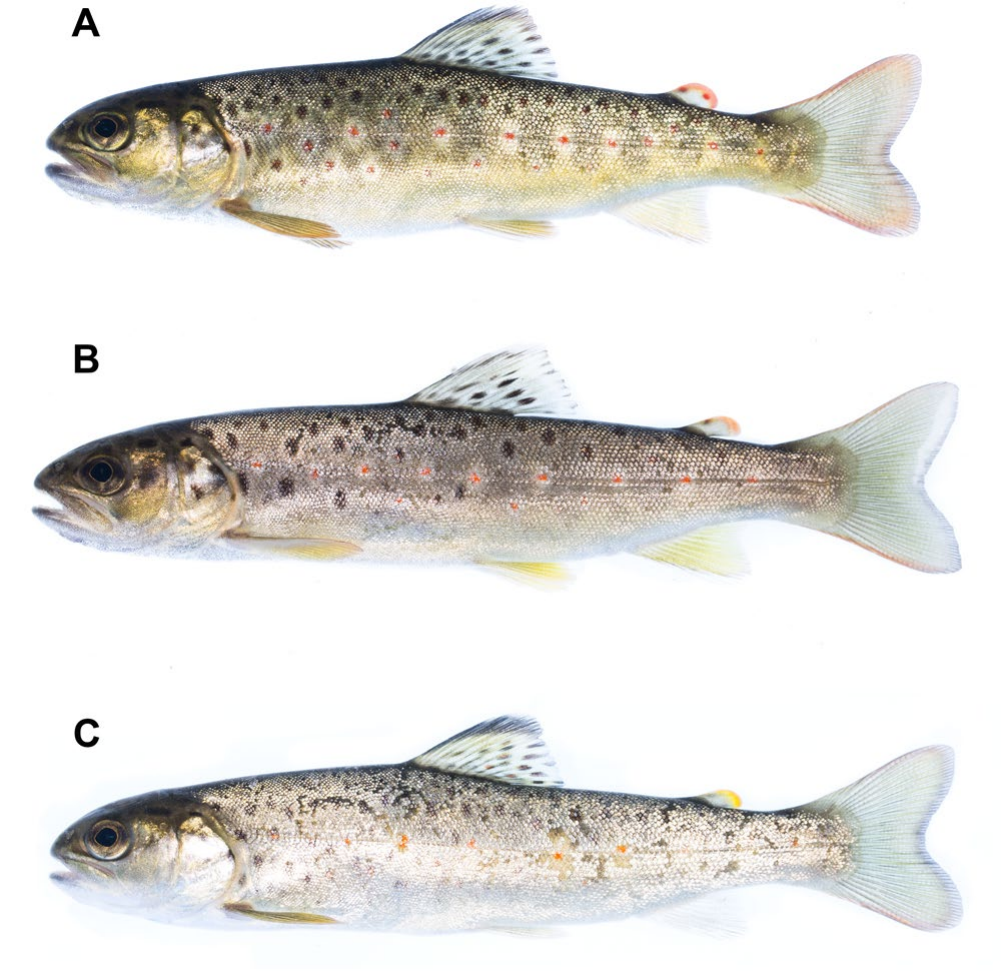
Representative morphology of the seaward-migrating trout classified as (**A**) parr, (**B**) pre-smolt, and (**C**) smolt.

The juvenile trout can enter saltwater at different stages during the parr to smolt transformation^20,24–27^. Based on their different external morphological features including body shape and coloration^20,24^, the migrating trout can be classified into three different phenotypes: parr, pre-smolts and smolts (Table 1; Fig. 1). Body colouration is an essential adaptation that allows the fish to blend into the surrounding environment, reducing the risk of being eaten by visual predators (e.g., birds and fish). For instance, dark coats and spotted sides allow parr to blend with bottom rocks and gravel in rivers. Conversely, the light bellies, silvery sides, and dark dorsal surface of smolts help camouflaging fish in a mid-water marine environment. In salmonids, there are also changes in body shape when smolts migrate to the sea in spring, which are likely associated with greater swimming efficiency, with the snout becoming more pointed, the body slimmer and more streamlined, and a lengthening of the caudal peduncle [e.g.^24,28,29^ Fig. 1]. Thus it could be hypothesised that when parr enter the sea, they are more vulnerable to predation than smolts as being less prepared for a marine life.

**Table 1:** Morphological characteristics of the seaward-migrating juvenile trout classified as parr, pre-smolt and smolt (after Tanguy^24^).

	Morphological characteristics
Parr	Light to yellowish body coloration, no silvering of scales, red spots, yellow to brownish-orange fin color, and parr marks are clearly present.
Pre-smolt	Parr marks almost completely obscured by the silvery appearance of the scales, receding red spots, and fish are relatively thinner in appearance than parr.
Smolt	Absence of parr marks, almost fully silvering of scales, no red spots, darkened fins, and fish are slender in appearance than parr and pre-smolts. The head is more pointed.

The different phenotypes of spring migrating juvenile trout may also differ in their capacity to osmoregulate in saltwater based on their different levels of gill Na^+^, K^+^-ATPase activity^20,21^. Osmoregulation in saltwater has been acknowledged as critical factor  for survival of diadromous fish at sea^19,20,24,30^. Hence, it can also be assumed that parr have lower survival at sea than smolts due to their lower capacity to osmoregulate in saltwater. In addition, migrant parr should stay in low saline water (e.g., fjord systems), whereas the more seawater pre-adapted pre-smolts and smolts should extend their migration beyond the fjord to exploit feeding areas at sea. However, little is known about the behaviour and survival of the various phenotypes of migrating trout once they enter the marine environments.

This study examines whether the different phenotypes of spring migrating juvenile trout (i.e., parr, pre-smolts and smolts) exhibit different life-history strategies (time of seaward migration and time spent at sea) and survival at sea based on the hypothesised differences in their degree of morphological and physiological adaptions for marine life. To test this, a total of 9297 wild juvenile trout were trapped in a Danish river in spring over two consecutive years, PIT-tagged, and categorized as parr, pre-smolts or smolts (based on body shape and coloration) during their downstream migration to the sea. Following tagging, their return from the sea to the river was monitored by a stationary PIT-antenna station, located close to the river mouth.

## Materials and Methods

### Study site

**Figure 2:**
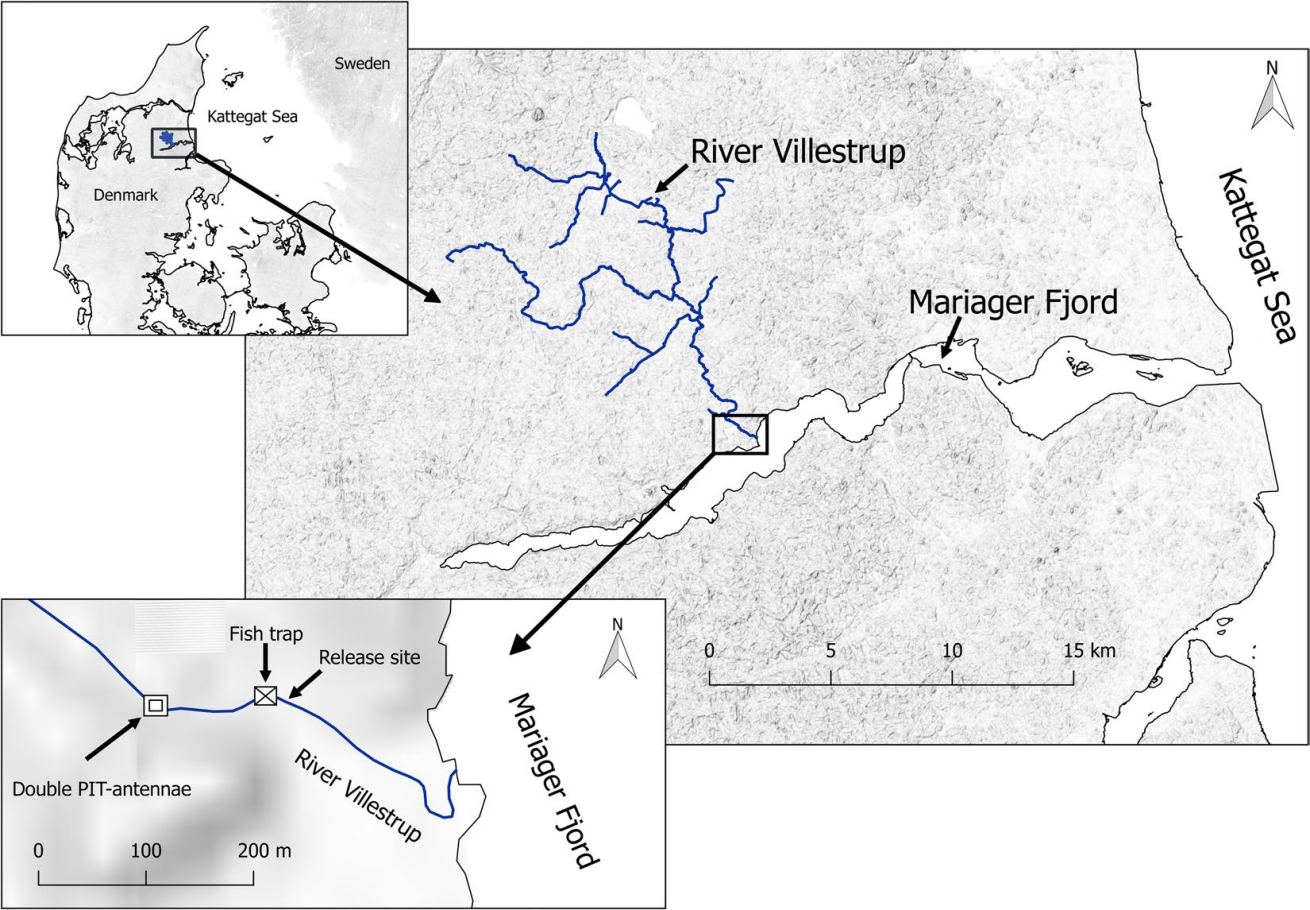
Mariager Fjord and River Villestrup on the east coast of Jutland, Denmark. The location of the trap, PIT-station and release site in River Villestrup are shown in the inset.

The River Villestrup is located on the east coast of Jutland, Denmark (Fig. 2). The river is about 20 km long and has a catchment area of 126 km^2^. The river flow is relatively stable due to large groundwater inflows with a mean annual discharge of 1.1 m^3^ s^−1^. The river discharges into Mariager Fjord, a sill fjord with relatively low water exchange with the Kattegat Sea^31,32^. The fjord is about 35 km long and 2 km wide with a total area of 47 km^2^. The fjord has a tidal range of 20–30 cm and salinity varies between 12 and 25‰ from the inner to the outer fjord.

The River Villestrup supports a population of partially anadromous brown trout, where a portion of the individuals smoltify and migrate to sea and the rest remain resident in the river. The proportion of migratory and resident trout in the river system is currently unknown. However, previous studies in Danish rivers typically show that more than two-thirds of the trout migrate to sea^33,34^. The river has not been stocked with hatchery-reared trout for the last four decades and the fish are therefore considered to be of wild origin^23^. In 2006, the population of sea trout spawners was estimated to be approximately 1200 fish and the average density of young of the year trout was estimated at 121 individuals per 100 m^2^ (95% CI: 31–211 individuals per 100 m^2^) in autumn 2007^35^.

### Fish trapping and tracking

A Wolf-type trap^36^ was used to capture fish during their downstream migration to the fjord. The trap was located 300 m from the river mouth and 100 m downstream from the PIT- antennae station (Fig. 2). The trap surface consisted of a grid of aluminium bars at 8 mm spacing, covering the whole cross-sectional area of the river. This type of trap is suitable for capturing trout larger than 100 mm in total length^20^ and it redirects fish into a holding chamber where they can be easily accessible. The trap was operated from the 31^th^ March to 2^nd^ June 2008 and from the 20^th^ March to 31^st^ May 2009. No overflow or malfunction occurred during the trapping periods.

All captured fish larger than 120 mm in total length were PIT-tagged (Texas Instruments, RI-TRP-RRHP, half duplex, 134 kHz, length 23.1 mm, diameter 3.85 mm and weight 0.6 g in air). The lower size limit of 120 mm was chosen to minimize possible tagging effects and tag losses^37^. Hence, it was not possible to include smaller migrating trout of a particular phenotype in this study. Approximately 12% of all trapped trout measured between 100–120 mm but the portion of migrating trout smaller than 100 mm remains unknown as they could swim through the trap. Fish tagging was conducted at the site of capture, the same day or the day following capture, to reduce handling and recovery time. Prior to surgical implantation of the tags, trout were anesthetized with benzocaine (25 mg/L, Sigma Chemical Co., St Louis, USA). Tagging was intraperitoneal via a small ventrolateral incision (3–4 mm long) posterior to the pectoral fins made with a scalpel. The tagged fish were placed in a recovery tank and released in the river downstream of the fish trap about one hour after tagging.

Tagged trout were classified as parr, pre-smolts or smolts based on external phenotypic characteristics (i.e., body shape and coloration) following Tanguy’s^24^ classification (see Table 1 and Fig. 1). This classification has been shown to be suitable for evaluating the developmental stage and osmoregulatory capacity of juvenile salmonids^5,21,22^. Additionally, total length to nearest millimetre and the PIT-tag number were registered.

A PIT-antennae station was deployed approximately 400 m upstream from the river mouth to register trout returning from the sea to the river (Fig. 2). This system continuously recorded date, time, and PIT-tag code of tagged fish passing the detection field. The station consisted of two swim-trough antennas located 10 m apart. The antennas were connected to stationary data-logging readers operating at 134 KHz (model TIRIS S- 2000, Texas Instruments). The paired use of antenna allows discrimination between up and downstream movements and increases the detection probability of tagged trout. The *in situ* detection efficiency of the PIT-station was calculated according to Zydlewski *et al*.^38^, and estimated at 98.5%. The PIT-station operated continuously from the beginning of the experiment in March 2008 until November 2012.

At the end of each trapping period in 2008 and 2009, electrofishing surveys were conducted in the river downstream of the trap to Mariager Fjord. None of the tagged trout were caught in this section of the river (i.e., last ~ 300 m of the river), suggesting that all tagged fish migrated to the fjord. Furthermore, the lower reach of the river has a limited number of suitable habitats for juvenile trout; the macrophyte vegetation is sparse and the bottom substrate consists mainly of sand. It is therefore unlikely that trout stayed in this river section as resident individuals. However, it was not possible to conclusively document that all trout entered the fjord system in the present study. In addition, it cannot be excluded that some parr and pre-smolts completed smoltification in the lower reaches of the river or Mariager Fjord. This could potentially make them belong to the smolt group later in the season.

### Use of experimental animals in this experiment

Trout were tagged under license (2012-DY-2934-00007) issued by the Danish Experimental Animal Committee. All animal tagging procedures were approved by the Danish Experimental Animal Committee in accordance with the guidelines described in the cited permission.

### Statistical analysis

#### Migration timing from the river to the sea

A GLM (generalized linear model) with a gamma distribution and log link function was used to test whether time of seaward migration, here defined as day of year an individual was captured and PIT-tagged (subsequently referred to as migration time), was related to fish group (parr, pre-smolt and smolt), year of tagging (2008 and 2009), and body length at tagging. The initial model included all two-way interactions:

$$\begin{array}{rll}{\rm{Migration}}\,{{\rm{time}}}_{{\rm{i}}} &  \sim  & {\rm{gamma}}({{\rm{\mu }}}_{{\rm{i}}},{\rm{\tau }})\\ {\rm{E}}({\rm{Migration}}\,{{\rm{time}}}_{{\rm{i}}}) & = & {{\rm{\mu }}}_{{\rm{i}}}\\ {\rm{var}}({\rm{Migration}}\,{{\rm{time}}}_{{\rm{i}}}) & = & \frac{{{\rm{\mu }}}_{i}^{2}}{{\rm{\tau }}}\end{array}$$

$$\mathrm{Log}({{\rm{\mu }}}_{{\rm{i}}})\,=\,{\rm{Intercept}}+{{\rm{Group}}}_{{\rm{i}}}+{{\rm{Year}}}_{{\rm{i}}}+{{\rm{Length}}}_{{\rm{i}}}+\,{{\rm{Group}}}_{{\rm{i}}}\times {{\rm{Year}}}_{{\rm{i}}}\,+\,{{\rm{Group}}}_{{\rm{i}}}\times {{\rm{Length}}}_{{\rm{i}}}\,+\,{{\rm{Year}}}_{i}\times {{\rm{Length}}}_{{\rm{i}}}$$

#### Return of sea trout to the river

The return rate of sea trout to River Villestrup was calculated as the number of fish that migrated back to the river and was detected by the PIT station relative to the total number of tagged fish. The ultimate fate of trout that did not return to the river could not be determined in the present study. Nonetheless, they may have been associated with any of these factors: fish mortality, tag loss by fish, non-detected fish by the PIT station, and fish straying to other rivers. Despite these limitations, the return rate of sea trout to the river can be used as a proxy for minimum survival at sea.

A GLM with a Bernoulli distribution and logit link function was used to assess the probability of fish return (yes/no) from the sea to River Villestrup in relation to fish group (parr, pre-smolt and smolt), year of tagging (2008 and 2009), length at tagging and migration time (day of year). All two-way interactions were included in the initial model:

$$\begin{array}{rll}{\rm{River}}\,{{\rm{return}}}_{{\rm{i}}} &  \sim  & {\rm{Bernoulli}}({{\rm{\pi }}}_{{\rm{i}}})\\ {\rm{E}}({\rm{River}}\,{{\rm{return}}}_{{\rm{i}}}) & = & {{\rm{\pi }}}_{{\rm{i}}}\\ {\rm{var}}({\rm{River}}\,{{\rm{return}}}_{{\rm{i}}}) & = & {{\rm{\pi }}}_{{\rm{i}}}\times ({\rm{1}}-{{\rm{\pi }}}_{{\rm{i}}})\end{array}$$

$$\begin{array}{rcl}{\rm{Logit}}({{\rm{\pi }}}_{{\rm{i}}}) & = & {\rm{Intercept}}+{{\rm{Group}}}_{{\rm{i}}}+{{\rm{Year}}}_{{\rm{i}}}+{{\rm{Length}}}_{{\rm{i}}}+{\rm{Migration}}\,{{\rm{time}}}_{{\rm{i}}}\\  &  & +\,{{\rm{Group}}}_{{\rm{i}}}\times {{\rm{Year}}}_{{\rm{i}}}+{{\rm{Group}}}_{{\rm{i}}}\times {{\rm{Length}}}_{{\rm{i}}}+{{\rm{Group}}}_{{\rm{i}}}\times {\rm{Migration}}\,{{\rm{time}}}_{{\rm{i}}}\\  &  & +\,{{\rm{Year}}}_{{\rm{i}}}\times {{\rm{Length}}}_{{\rm{i}}}+{{\rm{Year}}}_{{\rm{i}}}\times {\rm{Migration}}\,{{\rm{time}}}_{{\rm{i}}}+{{\rm{Length}}}_{{\rm{i}}}\times {\rm{Migration}}\,{{\rm{time}}}_{{\rm{i}}}\end{array}$$

#### Marine residence time

The marine residence time could only be determined for trout that returned to River Villestrup and was calculated as the number of days between tagging until first detection at the PIT-antenna station. The returning sea trout were divided into two different sea-age classes according to time spent at sea: 0SW (less than 365 days at sea) and 1 + SW (more than 365 days at sea).

We used a GLM with Bernoulli distribution and logit link function to investigate whether fish group (parr, pre-smolt and smolt), year of tagging (2008 and 2009), length at tagging, and migration time (day of year) were associated with sea-age class (0SW and 1 + SW) as a measure for marine residence time. All two-way interactions were included in the initial model:

$$\begin{array}{rll}{\rm{Sea}}-{\rm{age}}\,{{\rm{class}}}_{{\rm{i}}} &  \sim  & {\rm{Bernoulli}}({{\rm{\pi }}}_{{\rm{i}}})\\ {\rm{E}}({\rm{Sea}}-{\rm{age}}\,{{\rm{class}}}_{{\rm{i}}}) & = & {{\rm{\pi }}}_{{\rm{i}}}\\ {\rm{var}}({\rm{Sea}}-{\rm{age}}\,{{\rm{class}}}_{{\rm{i}}}) & = & {{\rm{\pi }}}_{{\rm{i}}}\times (1-{{\rm{\pi }}}_{{\rm{i}}})\end{array}$$

$$\begin{array}{rcl}{\rm{Logit}}({{\rm{\pi }}}_{{\rm{i}}}) & = & {\rm{Intercept}}+{{\rm{Group}}}_{{\rm{i}}}{+\mathrm{Year}}_{{\rm{i}}}+{{\rm{Length}}}_{{\rm{i}}}+{\rm{Migration}}\,{{\rm{time}}}_{{\rm{i}}}\\  &  & +\,{{\rm{Group}}}_{{\rm{i}}}\times {{\rm{Year}}}_{{\rm{i}}}+{{\rm{Group}}}_{{\rm{i}}}\times {{\rm{Length}}}_{{\rm{i}}}+{{\rm{Group}}}_{{\rm{i}}}\times {\rm{Migration}}\,{{\rm{time}}}_{{\rm{i}}}\\  &  & +{{\rm{Year}}}_{{\rm{i}}}\times {{\rm{Length}}}_{{\rm{i}}}+{{\rm{Year}}}_{{\rm{i}}}\times {\rm{Migration}}\,{{\rm{time}}}_{{\rm{i}}}+{{\rm{Length}}}_{{\rm{i}}}\times {\rm{Migration}}\,{{\rm{time}}}_{{\rm{i}}}\end{array}$$

#### Instantaneous mortality rate

The instantaneous mortality rate at sea (Z; day^−1^) was estimated for the three fish groups (parr, pre-smolt and smolt) in 2008 and 2009 as:

$${{\rm{N}}}_{{\rm{t}}}={{\rm{N}}}_{0}{{\rm{e}}}^{-{\rm{Zt}}}$$

where N_0_ is the number of trout tagged, N_t_ is the number of trout returning from the sea to the river, and t is the average number of days spent at sea. This allowed us to compare the time-based mortality rate at sea among the different phenotypes of trout. The equation assumes an exponential decline in the number of trout over time at sea.

#### General statistical notes

All statistical analyses were performed using R version 3.1.1^39^. Before applying the statistical models, data exploration was performed following the protocol in Zuur *et al*.^40^. Collinearity between covariates was assessed using generalized variance inflation factors prior to applying the models. All variance inflation factors were <2, suggesting negligible multicollinearity^41^. Model selection was conducted by backwards elimination using p = 0.05 as threshold for elimination. Model validation was performed by visual inspection of the residuals and no violations were encountered.

## Results

In 2008, a total of 4304 migrating juvenile trout were tagged and 4993 fish were tagged in 2009. The majority of the migrating trout were characterized as smolts (66.5%), followed by pre-smolts (29.6%) and parr (3.9%) (Table 2).

**Table 2:** Number of PIT-tagged trout in 2008 and 2009 classified as parr, pre-smolt and smolt, with the associated body length (mm) at tagging, migration time (day of year), return rate (%) to River Villestrup from the sea, time spent at sea (less [0 SW] or more [1 + SW] than one year), and instantaneous mortality rate (day^−1^) at sea.

	Number of tagged fish		Body length (mean and range)		Migration time (mean and range)		Return rate to River Villestrup		Time spent at sea		Instantaneous mortality rate	
	2008	2009	2008	2009	2008	2009	2008	2009	2008	2009	2008	2009
Parr	204 (4.7%)	155 (3.1%)	144.5 (120–271)	160.8 (120–285)	104.8 (91–140)	99.9 (79–143)	14.2% (29/204)	21.2% (33/155)	0SW (75.9%) 1 + SW (24.1%)	0SW (78.8%) 1 + SW (21.2%)	6.72 × 10^−3^	6.20 × 10^−3^
Pre-smolt	1229 (28.6%)	1522 (30.5%)	145.8 (120–235)	140.6 (120–262)	112.5 (91–153)	105.3 (79–143)	10.7% (132/1229)	10.4% (158/1522)	0SW (61.4%) 1 + SW (38.6%)	0SW (47.5%) 1 + SW (52.2%)	6.83 × 10^−3^	6.15 × 10^−3^
Smolt	2871 (66.7%)	3316 (66.4%)	156.0 (120–264)	150.7 (120–264)	116.6 (91–150)	114.0 (79–151)	6.9% (197/2871)	9.3% (307/3316)	0SW (48.2%) 1 + SW (51.8%)	0SW (47.9%) 1 + SW (52.1%)	6.82 × 10^−3^	6.42 × 10^−3^

### Migration timing from the river to the sea

Step-wise single term elimination of non-significant (p > 0.05) predictors resulted in the following final model:

$$\begin{array}{rll}{\rm{Migration}}\,{{\rm{time}}}_{{\rm{i}}} &  \sim  & {\rm{gamma}}({{\rm{\mu }}}_{{\rm{i}}},{\rm{\tau }})\\ {\rm{E}}({\rm{Migration}}\,{{\rm{time}}}_{{\rm{i}}}) & = & {{\rm{\mu }}}_{{\rm{i}}}\\ {\rm{var}}({\rm{Migration}}\,{{\rm{time}}}_{{\rm{i}}}) & = & \frac{{{\rm{\mu }}}_{i}^{2}}{{\rm{\tau }}}\end{array}$$

$$\begin{array}{rcl}\mathrm{Log}({{\rm{\mu }}}_{{\rm{i}}}) & = & {\rm{Intercept}}+{{\rm{Group}}}_{{\rm{i}}}+{{\rm{Year}}}_{{\rm{i}}}+{{\rm{Length}}}_{{\rm{i}}}\\  &  & +\,{{\rm{Group}}}_{{\rm{i}}}\times {{\rm{Length}}}_{{\rm{i}}}+{{\rm{Group}}}_{{\rm{i}}}\times {{\rm{Year}}}_{i}\end{array}$$

**Figure 3:**
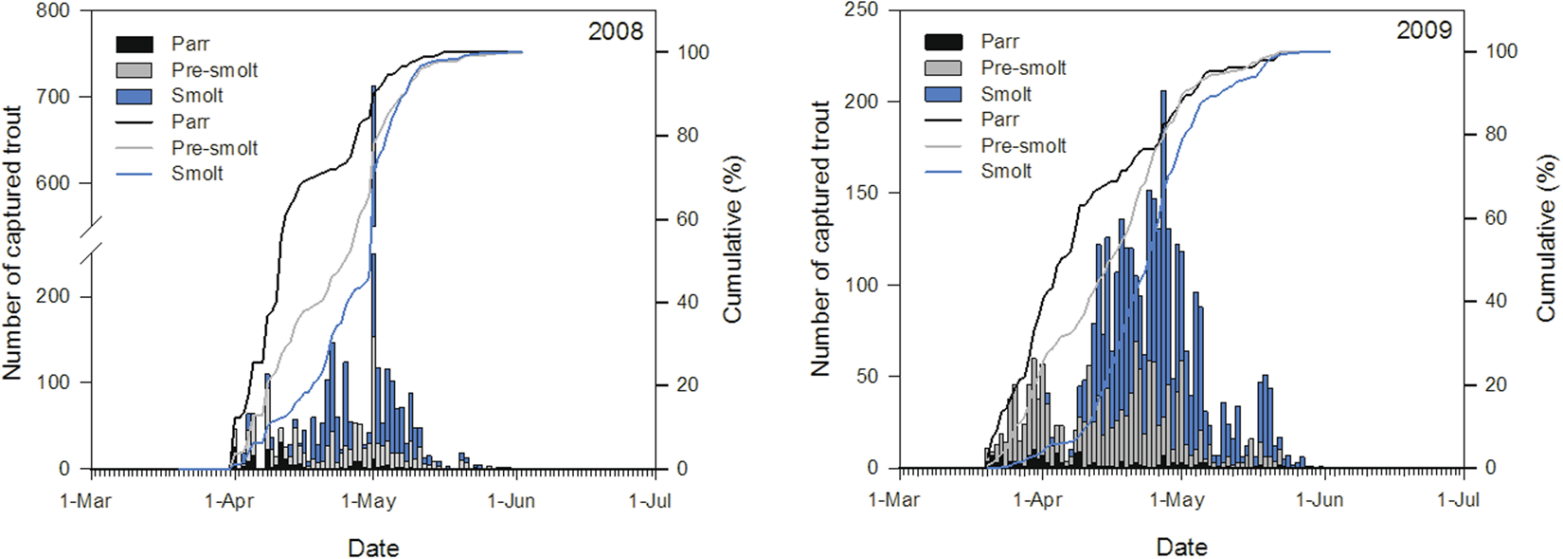
Number of PIT-tagged juvenile trout during the spring migration in 2008 (n = 4304) and 2009 (n = 4993). The solid lines represent the cumulative percentage of the migrating trout classified as parr (black line), pre-smolt (grey line) and smolt (blue line). The bars represent the daily number of migrating parr (black bars), pre-smolt (grey bars) and smolt (blue bars). Note the different scales on the y-axis.

Summary statistics of the final model are presented in Table 3. Trout were trapped migrating downstream from the end of March until end of May in both years and most fish (70%) migrated in April (Fig. 3). This period coincides with the normal time for the juvenile trout spring migration in the River Villestrup^16–18^.

**Table 3:** Summary statistics of the final model describing the effect of Group, Year and Length on the time of seaward migration for the juvenile trout.

Source of variation	F	df	p
Group	451.81	2	<0.0001
Year	312.91	1	<0.0001
Length	42.12	1	<0.0001
Group × Length	57.50	2	<0.0001
Group × Year	34.90	2	<0.0001

**Figure 4:**
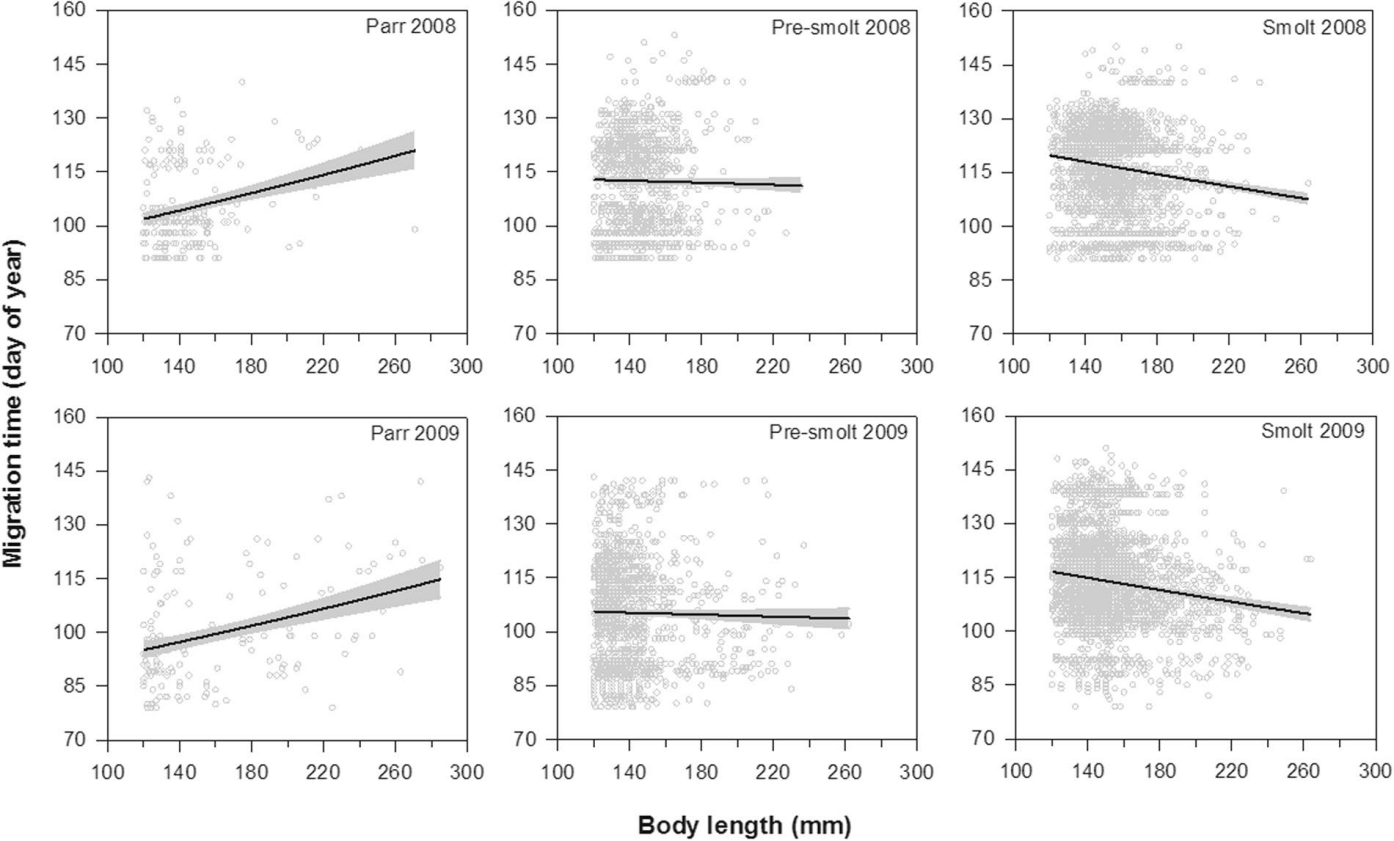
Relationship between migration time (day of year) from River Villestrup to the sea and body length (mm) of trout classified as parr, pre-smolt and smolt in 2008 and 2009. Solid lines represent the model fit and shaded areas represent 95% confidence intervals. Open circles indicate observed values. See Table 2 for sample sizes.

All phenotypes of trout were captured in the river throughout the trapping period in 2008 and 2009 (Fig. 3). However, parr generally migrated earlier compared to pre-smolt and smolts in both years, and smolts tended to migrate later than pre-smolts (GLM: Group × Year, F = 34.90, df = 2, p < 0.0001; Figs 3 and 4, Table 2). It should be stressed that the interaction between Group and Year is barely noticeable on Fig. 4, despite being statistically significant (Table 3). Therefore, the interactive effect between Group and Year on the migration timing should be interpreted with great care.

In addition, time of seaward migration for the different phenotypes of trout was associated with body length at tagging (GLM: Group × Length, F = 57.50, df = 2, p < 0.0001; Fig. 4). While body length was positively correlated with migration time for parr, a negative relationship was found for smolts. For pre-smolts, there was no clear relationship between body length and timing of migration (Fig. 4).

### Return of sea trout to the river

Step-wise single term elimination of non-significant (p > 0.05) predictors resulted in the following final model:

$$\begin{array}{c}\begin{array}{l}{\rm{River}}\,{{\rm{return}}}_{{\rm{i}}} \sim {\rm{Bernoulli}}({{\rm{\pi }}}_{{\rm{i}}})\\ {\rm{E}}({\rm{River}}\,{{\rm{return}}}_{{\rm{i}}})={{\rm{\pi }}}_{{\rm{i}}}\end{array}\\ {\rm{var}}({\rm{River}}\,{{\rm{return}}}_{{\rm{i}}})={{\rm{\pi }}}_{{\rm{i}}}\times (1-{{\rm{\pi }}}_{{\rm{i}}})\end{array}$$

$$\begin{array}{rcl}{\rm{Logit}}\,({{\rm{\pi }}}_{{\rm{i}}}) & = & {\rm{Intercept}}+{{\rm{Group}}}_{{\rm{i}}}+{{\rm{Year}}}_{{\rm{i}}}+{{\rm{Length}}}_{{\rm{i}}}\\  &  & +{\rm{Migration}}\,{{\rm{time}}}_{{\rm{i}}}+{{\rm{Group}}}_{{\rm{i}}}\times {{\rm{Length}}}_{{\rm{i}}}\end{array}$$

**Figure 5:**
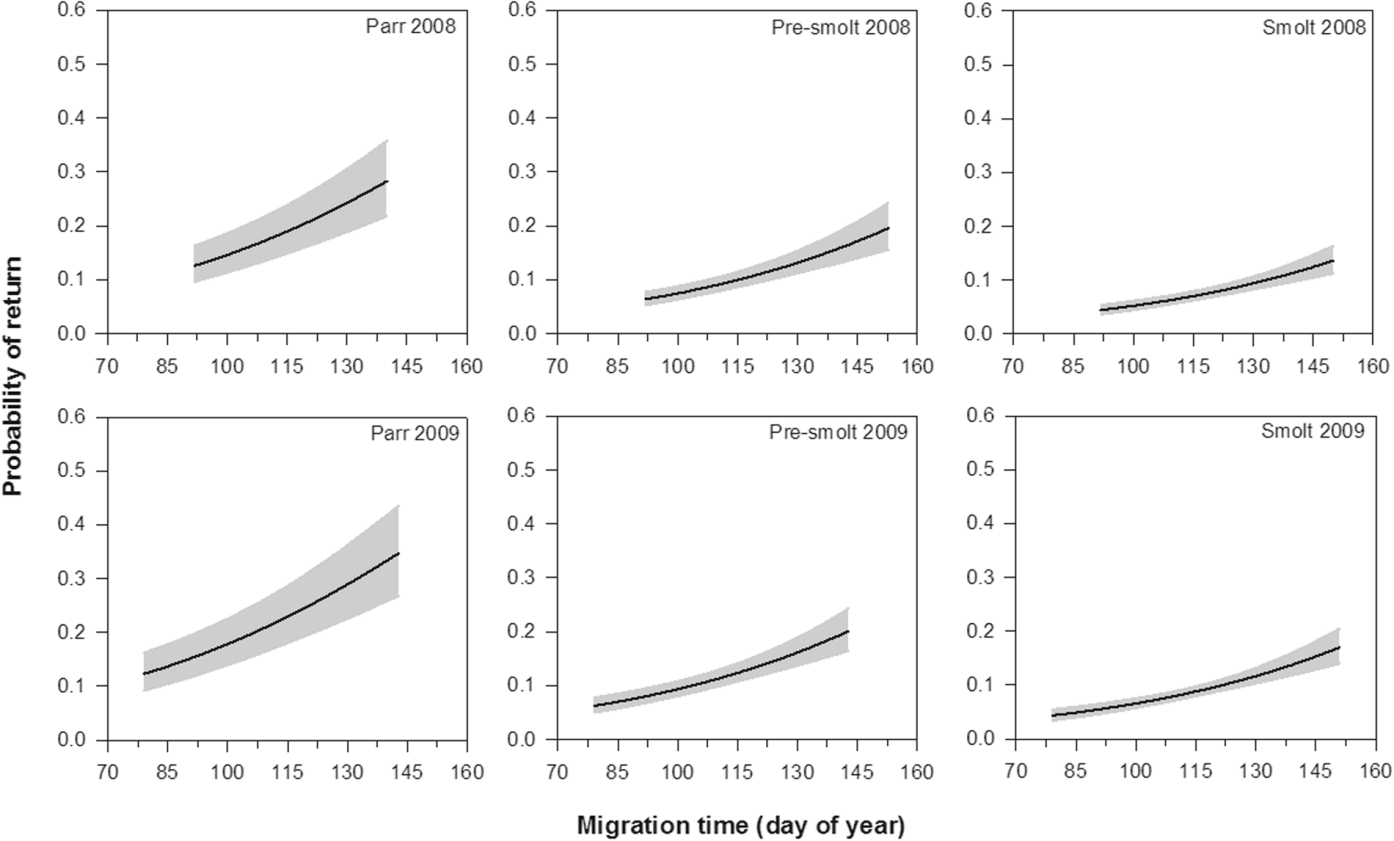
Probability of return to River Villestrup for sea trout in relation to migration time (day of year) for the seaward-migrating trout classified as parr, pre-smolt and smolt in 2008 and 2009. Solid lines represent the predicted probability of return and shaded areas indicate 95% confidence intervals. Body length was set to the average value of 150.1 mm.

**Figure 6:**
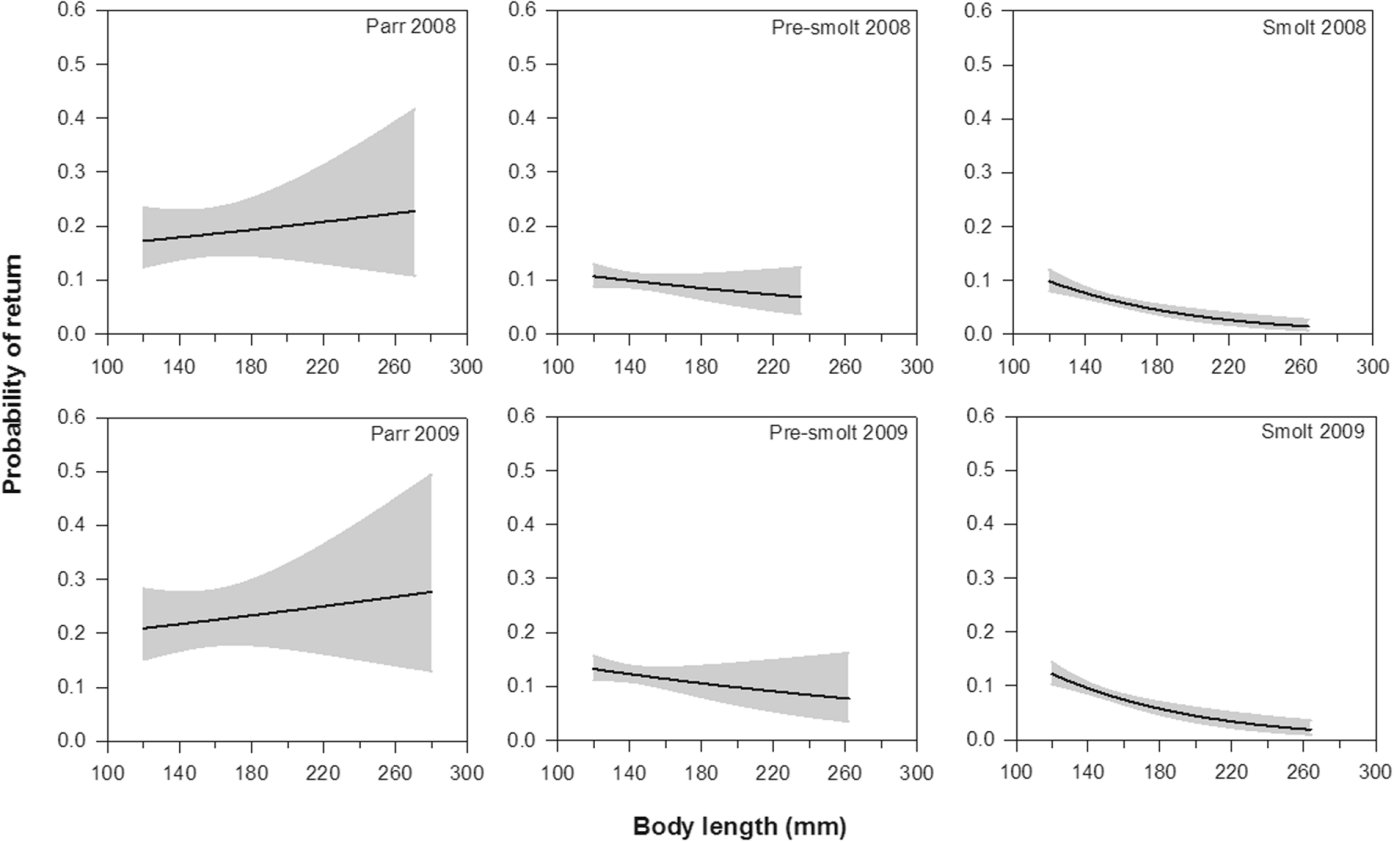
Probability of return to River Villestrup for sea trout in relation to body length (mm) at tagging for the seaward-migrating trout classified as parr, pre-smolt or smolt in 2008 and 2009. Solid lines represent the predicted probability of return and shaded areas indicate 95% confidence intervals. Migration timing was set to the average value of 112.7 days.

Summary statistics of the final model are presented in Table 4. The probability of return for sea trout to River Villestrup differed significantly between years: individuals tagged in 2008 had lower return rate than those tagged in 2009 (GLM: Year, LRT = 10.15, df = 1, p = 0.001). The overall return of sea trout was 8.3% (n = 358 out of 4304) and 10.0% (n = 498 out of 4993) in 2008 and 2009, respectively. The model also showed that the probability of return was higher for trout that migrated later in the season (GLM: Migration time, LRT = 50.53, df = 1, p < 0.0001; Fig. 5). Lastly, body length at tagging had a significant effect on the probability of return among the fish groups (GLM: Group × Length, LRT = 12.30, p = 0.002; Fig. 6). In the groups of smolt and pre-smolt, the probability of return was greater for smaller individuals, while no conclusive effect was observed for parr (see Fig. 6). Notably, the probability of return was higher for parr (the return rate was 14.2% and 21.2% in 2008 and 2009, respectively) than that of pre-smolt (10.7% and 10.4%) and smolt (6.9% and 9.3%; Table 2; Fig. 6).

**Table 4:** Summary statistics of the final model describing the effect of Group, Year, Length, and Migration time on the probability of return of trout from the sea to River Villestrup.

Source of variation	LRT	df	p
Group	55.88	2	<0.0001
Year	10.15	1	0.001
Length	15.58	1	<0.0001
Migration time	50.53	1	<0.0001
Group × Length	12.30	2	0.002

#### Marine residence time

Step-wise single term elimination of non-significant (p > 0.05) predictors resulted in the following final model:

$$\begin{array}{c}\begin{array}{l}{\rm{Sea}} \mbox{-} {\rm{age}}\,{{\rm{class}}}_{{\rm{i}}} \sim {\rm{Bernoulli}}\,({{\rm{\pi }}}_{{\rm{i}}})\\ {\rm{E}}({\rm{Sea}} \mbox{-} {\rm{age}}\,{{\rm{class}}}_{{\rm{i}}})={{\rm{\pi }}}_{{\rm{i}}}\end{array}\\ {\rm{var}}({\rm{Sea}} \mbox{-} {\rm{age}}\,{{\rm{class}}}_{{\rm{i}}})={{\rm{\pi }}}_{{\rm{i}}}\times (1-{{\rm{\pi }}}_{{\rm{i}}})\end{array}$$

$$\begin{array}{rcl}{\rm{Logit}}({{\rm{\pi }}}_{{\rm{i}}}) & = & {\rm{Intercept}}+{{\rm{Group}}}_{{\rm{i}}}+{{\rm{Year}}}_{{\rm{i}}}+{{\rm{Length}}}_{{\rm{i}}}\\  &  & +\,{\rm{Migration}}\,{{\rm{time}}}_{{\rm{i}}}+{{\rm{Year}}}_{{\rm{i}}}\times {{\rm{Length}}}_{{\rm{i}}}\end{array}$$

**Figure 7:**
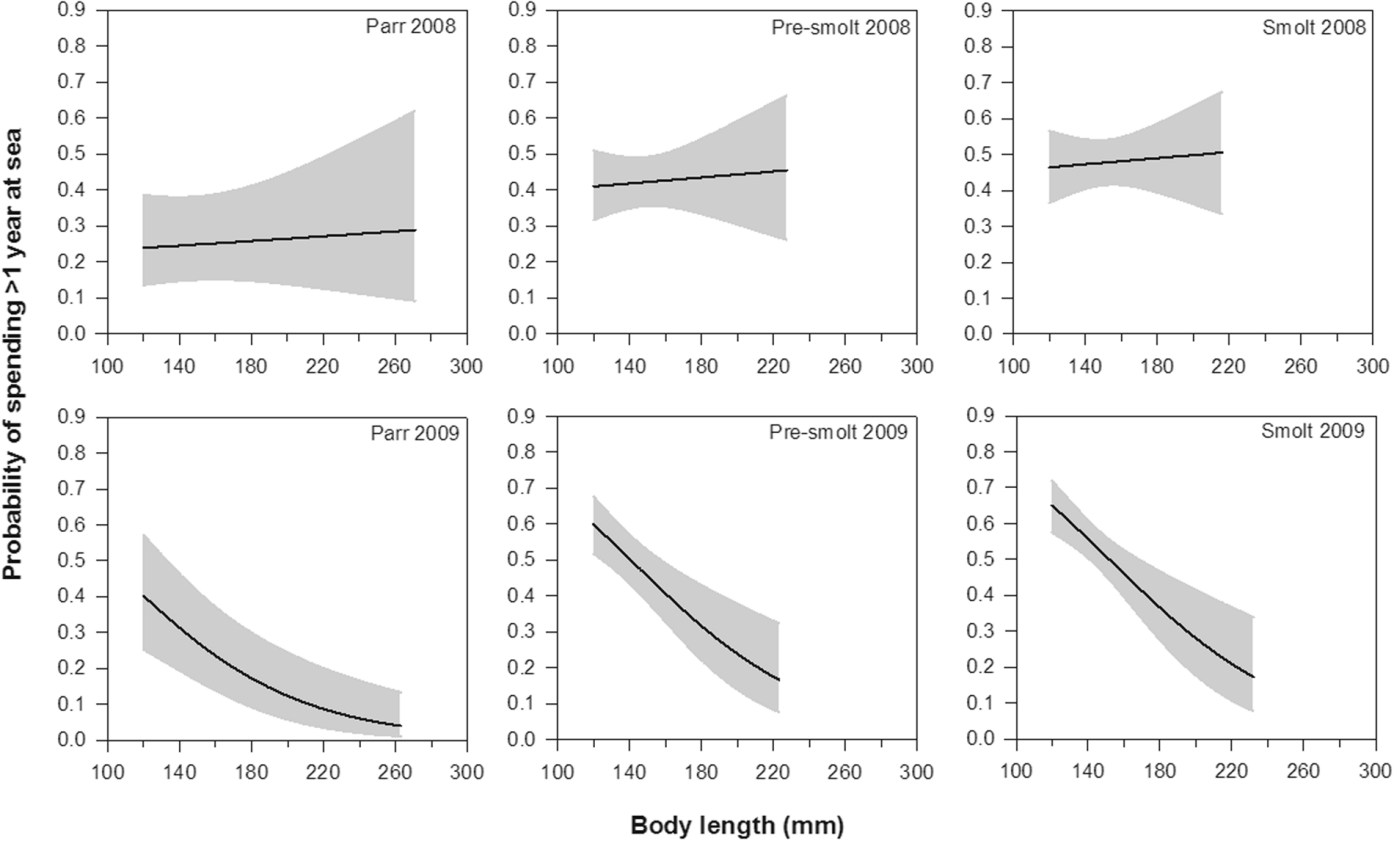
Probability that sea trout spent more than one year at sea before returning to River Villestrup in relation to body length (mm) at tagging for the seaward-migrating trout classified as parr, pre-smolt and smolt in 2008 and 2009. Solid lines represent the predicted probability of return and shaded areas indicate 95% confidence intervals. Migration time was set to the average value of 114.9 days.

**Figure 8:**
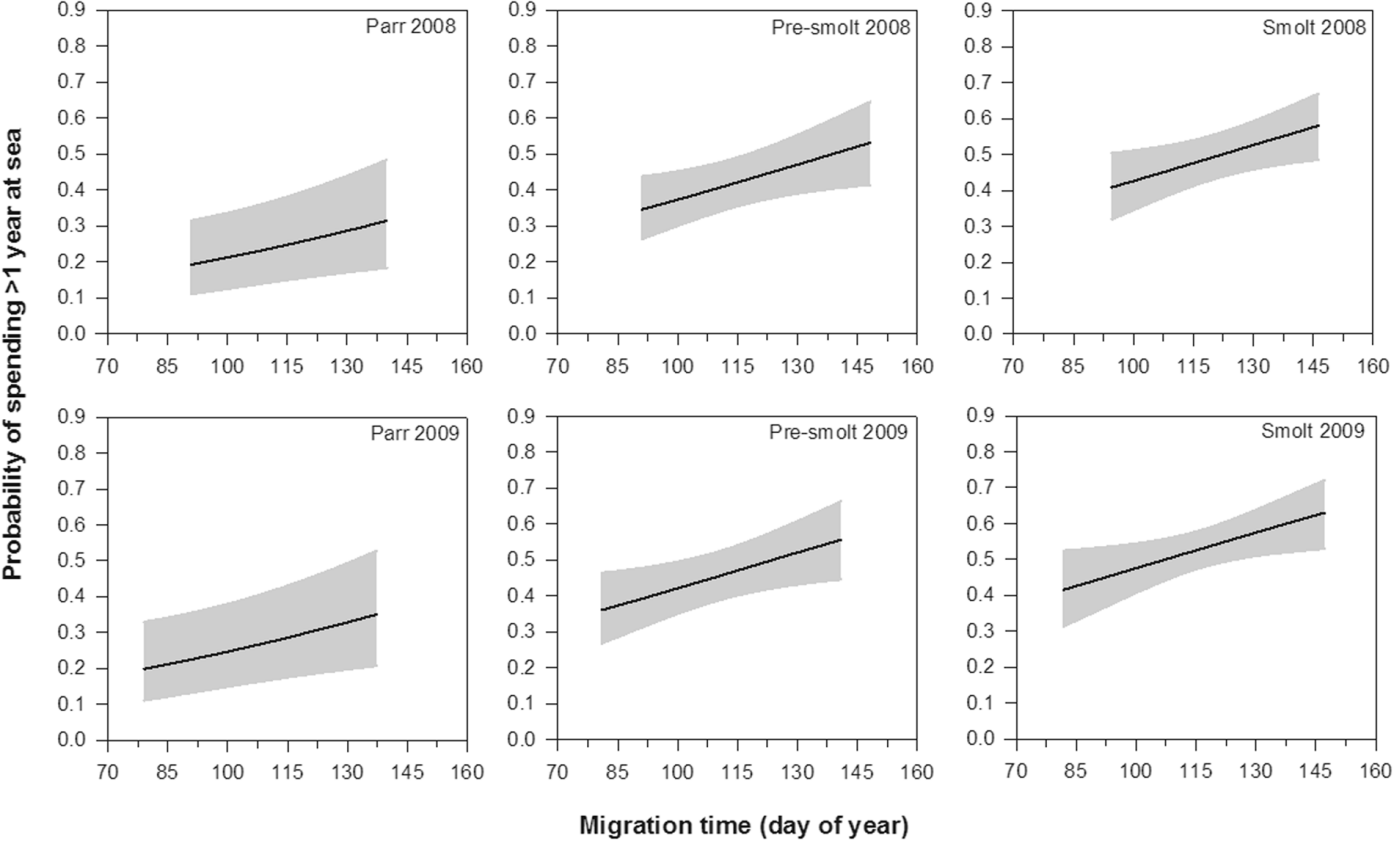
Probability that sea trout spent more than one year at sea before returning to River Villestrup in relation to migration time (day of year) for the seaward-migrating trout classified as parr, pre-smolt and smolt in 2008 and 2009. Solid lines represent the predicted probability of return and shaded areas indicate 95% confidence intervals. Body length was set to the average value of 146.6 mm.

Summary statistics of the final model are presented in Table 5. The time spent at sea differed significantly among fish groups (GLM: Group, LRT = 11.13, df = 2, p = 0.004; Table 2, Fig. 7). Tukey contrast test using the “glht” function in the “multcomp” package^42^ showed that parr had higher probability of staying less than one year at sea than pre-smolts (p = 0.043) and smolts (p = 0.005). In fact, 77% of the tagged parr that returned to the river did so within the first year following tagging, while 54% and 45% of the returning pre-smolts and smolts spent less than one year at sea, respectively (Table 2). In addition, smaller trout tagged in 2009 had higher probability of staying more than one year at sea compared to larger individuals, whereas no effect of body length on marine residence time was observed among trout tagged in 2008 (GLM: Year × Length, LRT = 8.75, df = 1, p = 0.003; Fig. 7). The model also showed that time of seaward migration affected time spent at sea, with trout migrating later having greater propensity to stay longer at sea (GLM: Migration time, LRT = 5.38, df = 1, p = 0.020, Fig. 8).

**Table 5:** Summary statistics of the final model describing the effect of Group, Year, Length, and Migration time on the probability that trout spent less or more than 1 year at sea before returning to River Villestrup.

Source of variation	LRT	df	p
Group	11.13	2	0.004
Year	1.81	1	0.178
Length	8.23	1	0.004
Migration time	5.38	1	0.020
Year × Length	8.75	1	0.003

#### Instantaneous mortality rate

The instantaneous mortality rate at sea was 6.72 × 10^−3^, 6.83 × 10^−3^ and 6.82 × 10^−3^ day^−1^ for parr, pre-smolts and smolts tagged in 2008, respectively (Table 2). For parr, pre-smolts and smolts tagged in 2009 the instantaneous mortality rate at sea was respectively 6.20 × 10^−3^, 6.15 × 10^−3^ and 6.42 × 10^−3^ day^−1^.

## Discussion

### Migration timing from the river to the sea

During the period from March to June in 2008 and 2009, wild sea trout juveniles migrated from the river and likely entered the sea at different developmental stages during their parr-smolt transformation.

The majority of the spring migrants were characterized as smolts (66.5%) followed by pre-smolts (29.6%) and parr (3.9%). Trout classified as parr generally migrated earlier to the sea than pre-smolt and smolts. This suggests that parr phenotypes were functionally adapted to life at sea and migrated downstream without having completed smoltification, at least with regard to changes in body morphology and coloration (i.e. silvery body, darkened fins and slim body shape). This phenomenon has previously been observed in other investigations^26,43^. Movement of parr to marine environments is also known to occur in autumn and winter in this river and others^44–46^. The migration of parr to the sea may reflect a distinctive life history strategy by which fish achieve better growth in hypotonic estuaries and coastal areas like Mariager Fjord than in their home river. This assumption has also been proposed in other studies involving salmonids (reviewed in^47^). However, an important limitation of the present study is that the sea trout were not recaptured in the river (e.g. by electrofishing) once they returned. Furthermore, juvenile trout that remained resident in the river were not included in this study. Without such data the costs and benefits of migrating to the sea versus staying in the river remain speculative.

The relationship between individual’s initial body length and the time they migrated to the sea showed different patterns among the phenotypes. Smolts followed the general trend with larger individuals migrating earlier in the season compared to smaller ones^48,49^. This behaviour is often explained by the lower predation risk associated with larger fish sizes as well as their reduced energetic costs during the seaward migration^50,51^. The opposite pattern was found for parr with smaller fish migrating earlier than larger individuals and body length of pre-smolts did not influence time of seaward migration. The mechanisms underlying these migratory differences remain unknown and highlight the need for additional studies on anadromous trout populations.

### Return of sea trout to the river

Sea trout classified as parr at time of tagging had a higher return rate to the river compared with pre-smolts and smolts, suggesting that the degree of pre-adaptation to saltwater is not of overriding importance for the survival of trout in Mariager Fjord. These results add to the growing body of evidence that trout may not need to complete smoltification to survive in marine environments for extended periods of time, at least not in low saline areas^25–27,43,52^. This possibility is critical for studies investigating the migratory behaviour and survival of trout at sea. For instance, considering merely individuals with silvery body coloration and absence of parr marks as true sea trout smolts risks excluding the contribution made by migrant parr and pre-smolt to the anadromous population (up to 34% in the present study; Table 2). Potentially, this might bias estimates of sea trout population sizes in the rivers and marine survival, for instance, when evaluating sea survival as the number of returning adults in relation to the number of seaward migrating smolts. However, it should be stressed that parr phenotypes only constituted 3.9% of the total numbers of tagged fish in the present study, and consequently their importance for the whole trout population might be limited. Lastly, it cannot be ruled out that parr tolerated handling and tagging better than fully smoltified trout, which may have contributed to the higher return rate of parr to the river.

The return from the sea to the river was lower for larger fish among pre-smolts and smolts, but no clear pattern was found for parr. Since predation has been recognized as one of the primary factors affecting survival of juvenile trout in similar fjord systems around Denmark^9,10^, this result may suggest a size selective predation biased towards larger fish sizes. This interpretation however, is in contrast to several other studies showing reduced predation risk as body size increases (reviewed in^50^). Hence, other confounding factors could account for the generally lower marine survival of larger individuals. Numerous studies have documented how habitats providing faster growth often have a higher predation risk than habitats supporting slower growth (e.g.^51,53^). This requires fish to make a behavioural trade-off between rapid growth and protection from predators. Thus, for sea trout populations distributed across a mosaic of habitats, as is the case for trout in the current system^54^, bigger might be better within a single habitat, but not necessarily across the population when considering that many fast growing individuals reside in risky habitats.

The return rate to the river was higher for trout that migrated later to the sea. This is in agreement with the findings of several previous studies on sea trout smolts [e.g.^48,49,54–56^] and numerous mechanisms could explain this pattern. For instance, food availability and predation pressure often fluctuate in fjord systems during the smolt season and have been recognised as important factors influencing the survival of trout in the early marine phase^14,57^. It is therefore possible that the early migrating trout experienced higher predation rates (e.g., from birds) and/or lower food availability compared to conspecifics migrating later to the sea. Parasitism is another potential factor influencing trout survival e.g.^58^, and if early migrants were more exposed or susceptible to parasites this could result in increased mortality. Furthermore, recent research has shown that juvenile trout can shift stream system through marine environments^59^. Hence, the lower return rate of early migrants could also be due to a higher migration rate of these individuals into non-natal streams in the fjord system. Additional telemetry studies are required to validate these assumptions.

### Marine residence time

77% of the returning parr spent less than one year at sea. This differs from the other phenotypes whereby 54% of pre-smolts and 45% of smolts returned from the sea after less than one year. The greater proportion of parr returning to the river after less than a year may suggest they remained in the fjord close to the river of origin. Conversely, the lower proportion of pre-smolts and smolts returning after one year may reflect a migration out of the fjord to the sea for these phenotypes, possibly to explore other feeding habitats (e.g. in the Kattegat Sea). This scenario is supported by a parallel study demonstrating that sea trout show different migratory strategies in Mariager Fjord (i.e. ranging from fjord residents to individuals migrating out of the fjord)^54^. Indeed, recent evidence suggests that sea trout from the same population can display high variation in habitat utilization and migration behaviour in fjord systems^15,57,60^. The PIT-telemetry methods used in this study did not allow us to assess the extent of migration out of the fjord system. Other methods, such as acoustic telemetry or otolith microchemical analysis, would be useful in addressing this question and further exploring the marine behaviour and habitat use of different trout phenotypes.

As previously mentioned, parr phenotypes were found to have a relatively higher return rate to the River Villestrup than the other phenotypes. In an attempt to clarify the mechanism underlying the greater return rate of parr to the river, instantaneous mortality rates at sea were calculated for each of the phenotypes. The results indicated similar daily mortality rates for each phenotype, suggesting that parr, pre-smolts and smolts performed equally at sea. Hence, the higher return rate of parr compared to pre-smolts and smolts seems to be related to their shorter duration at sea.

Trout that migrated earlier to the sea had a higher probability of staying less than one year at sea than conspecifics migrating later in the season. Early downstream migration prolongs the first growing season at sea and could potentially increase an individual’s chances of reaching sexual maturity at the end of the season. Thus, it is possible that a higher proportion of the early migrating trout reached sexual maturity at the end of their first marine season in comparison to fish migrating later and therefore returned earlier from the sea to the river to spawn. However, more research is needed to confirm this possibility. The relationship between time of seaward migration and marine residence time deserves more attention in future studies.

Lastly, the body length of trout tagged in 2009 was negatively correlated with time spent at sea. Although the explanation for this relationship remains unknown, it is possible that smaller trout can achieve a greater fitness by staying longer at sea relative to larger individuals as also seen in other salmonid species^61,62^. Again, recapture of adult trout in the river with known time at sea and size at tagging is needed to validate this possibility. The marine residence time was not influenced by body length of the trout tagged in 2008, emphasising that conclusions of behavioural studies on sea trout should ideally be drawn on data collected over multiple years.

Sea trout display a wide range of life-histories and after feeding in marine environments they can return to the natal river as immature or mature individuals for wintering and/or spawning^23,24,43,62,63^. It is important to note that the return rate of immature and mature trout from the sea to the riverine environment is not directly comparable, at least not in the context of survival until first sexual reproduction. Unfortunately, the proportion of immature and mature trout returning from the sea could not be determined in the present study as the river was not resampled during the spawning season. However, the return rate estimates for the different phenotypes likely included both immature and mature trout. Keeping in mind that 77% of the returning parr spent less than one year at sea, it is possible that a higher proportion of the parr used the fjord as summer habitat and returned to the river as immature trout for wintering compared to the other phenotypes. Conversely, the longer duration at sea for pre-smolts and smolts may suggest that a higher proportion returned to the river as mature trout for spawning. Thus, while parr had surprisingly high return rate to the river, their marine survival might not be directly comparable with the survival of pre-smolts and smolt at the time of first return to the river. The return rates of the phenotypes should therefore be compared with caution.

## Conclusion

In accordance with previous studies, the results of the present study suggest that the various phenotypes of seaward-migrating juvenile trout not only differ in morphology, but also display different life-history strategies in terms of migration time to the sea, marine residence time and potentially habitat use. It is suggested that these differences in life-history strategies can influence survival at sea. In particular, the relative higher return rate to the river for parr compared to pre-smolts and smolts seems to be related to their shorter duration at sea and/or potential difference in habitat utilization.

In the present study, trout were divided into the three phenotypic groups based on visual assessment of body shape and coloration. Although this approach has also been used in previous studies, image analysis of body morphometric and coloration provides unbiased and quantitative data with high reproducibility. Quantitative imaging techniques should therefore be considered in future studies as a tool for grouping trout with distinct phenotypic traits. Furthermore, development of a continuous classification system, potentially including physiological measurements coupled with fine scale telemetry or otolith microchemistry, might more fully resolve how different levels of seawater preparedness during the downstream spring migration affects behaviour and survival at sea. Future studies should also attempt to compare fitness-oriented endpoints (e.g., growth and fecundity) among the different phenotypes of seaward-migrating trout once they return to the river. This would allow an in-depth assessment of the costs and benefits of long versus short time at sea and migrating earlier versus later to the sea.
